# Social connection as a public health adaptation to extreme heat events

**DOI:** 10.17269/s41997-020-00309-2

**Published:** 2020-03-16

**Authors:** Amani Kafeety, Sarah B. Henderson, Amy Lubik, Jesse Kancir, Tom Kosatsky, Michael Schwandt

**Affiliations:** 1 Environmental Health Services, BC Centre for Disease Control, Vancouver, BC Canada; 2 Faculty of Medicine, School of Population and Public Health, University of British Columbia, Vancouver, BC Canada; 3 Population and Public Health, Fraser Health Authority, Surrey, BC Canada; 4 National Collaborating Centre for Environmental Health, Vancouver, BC Canada

**Keywords:** Social isolation, Social connection, Climate change, Extreme heat, Older adults, Isolement social, Lien social, Changement climatique, Chaleur extreme, Aînés

## Abstract

Climate change is an increasingly important public health issue, reflected in morbidity and mortality outcomes during extreme heat events. At the same time, the harms of social isolation with respect to a wide range of health outcomes are becoming better understood. Given that older adults are at higher risk during hot weather and at higher risk of social isolation, they are among those at highest risk for adverse impacts of extreme heat events. While specific strategies to reduce heat exposure have been described in the literature and promoted in public health practice, these may not be readily available to socially isolated older adults. As such, it is crucial to identify key approaches to address risk due to social isolation in the aging population, and to acknowledge their limitations and barriers. Interventions rooted in social connection, a concept widely applied in interventions for public health and social well-being, should be applied as a tool for adaptation to extreme heat events.

## Résumé

Le changement climatique est un problème de santé publique de plus en plus important, qui se reflète dans les résultats de la morbidité et de la mortalité lors des épisodes de chaleur extrême. Au même temps, les mals de l'isolement social en ce qui concerne un large éventail de résultats pour la santé sont de mieux en mieux compris. Étant donné que les personnes âgées sont plus à risque durant les temps chaud et à risque d'isolement social, elles font partie des personnes les plus exposées aux effets négatifs d'événements de chaleur extrême. Bien que des stratégies spécifiques pour réduire l'exposition à la chaleur aient été décrites dans la littérature et promues dans la pratique de la santé publique, elles peuvent ne pas être facilement accessibles aux personnes âgées socialement isolées. Comme tel, il est essentiel d'identifier les approches clés pour réduire les risques dus à l'isolement social de la population vieillissante et de reconnaître leurs limites et obstacles. Les interventions enracinées dans la connexion sociale, un concept largement appliqué dans les interventions pour la santé publique et le bien-être social, devraient être appliquées comme un outil d'adaptation aux événements de chaleur extreme.

## Climate change and the health impact of extreme heat events

“Extreme exogenous factors such as the climate have become disastrous partly because the emerging isolation and privatization, the extreme social and economic inequalities, and the concentrated zones of affluence and poverty pervasive in contemporary cities create hazards for vulnerable residents in all seasons.” (Klinenberg 2002, p. 230)

The surface temperature of the earth is increasing, with increases in the frequency and intensity of extreme heat events across the globe. Over the next several decades, Canada is predicted to increasingly experience days with temperatures over 30 °C, which are strongly associated with heat-related population mortality (Austin et al. 2016). In 2009, an extreme heat event in greater Vancouver caused an estimated 110 deaths (Kosatsky et al. 2012); in 2010, another event in Montreal caused an estimated 106 deaths (Price et al. 2013). Without more effective intervention, the burden of death and severe illness due to extreme heat events in Canada is predicted to rise in the coming years (Cheng et al. 2008).

## Social isolation, older adults, and vulnerability to extreme heat

The population of adults over age 65 in Canada is growing at a rate of 3.5% annually, which is approximately four times the rate of general population growth (NSC 2017). A growing population of older adults includes a concomitantly growing group of at-risk individuals prone to particular social and environmental risks. Among these risks, older adults are especially sensitive to the effects of heat (Arthurson and Baum 2015; Kilbourne 2002; Lubik and Kosatsky 2019; Yardley et al. 2011). While this is partially due to the increased burden of chronic disease and decreased thermoregulation associated with aging, increased social isolation among older adults is an important consideration when assessing impact of heat events (Arthurson and Baum 2015; Kilbourne 2002; Lubik and Kosatsky 2019; Yardley et al. 2011). Specifically, social isolation was identified as a key risk factor for mortality during both the 1995 heat event in Chicago (Klinenberg 2002) and the 2003 heat event in Paris (Poumadère et al. 2005). In both cases, socially isolated older adults were far more likely to experience adverse impacts from extreme heat than their socially integrated counterparts (Arthurson and Baum 2015; Kilbourne 2002; Lubik and Kosatsky 2019; Yardley et al. 2011). These noteworthy connections between social isolation, extreme heat events, and vulnerability of the older adult population necessitate more effective interventions.

Social isolation occurs when individuals are disconnected from social networks and supports, including resources for health protection and health promotion. The concept of social isolation is complex and relates to other constructs such as loneliness, social vulnerability, social inclusion and exclusion, social connectedness, and social capital. Social isolation can be defined as an objective situation in which an individual has a low quantity or quality of contact with others, and may include a paucity of “feeling of belonging, fulfilling relationships, and engagement with others” (National Seniors Council 2017).

Social isolation is a critical problem because it impedes the human ability to belong and to have healthy self-esteem. People living in social isolation often suffer from stress, anxiety, and depression, which can trigger adverse mental health outcomes that affect their coping ability during emergencies (Lubik and Kosatsky 2019). In addition, social isolation has been identified as a risk factor for a wide range of acute and chronic physical conditions (Arthurson and Baum 2015; Yardley et al. 2011). The impact of social isolation on health may be particularly severe for populations experiencing other forms of marginalization and vulnerability, such as Indigenous people, new immigrants, the economically disadvantaged, the homeless, and older adults (Lubik and Kosatsky 2019; Nurse et al. 2010). According to a report from the National Seniors Council (2017), approximately 16% of Canadian older adults are socially isolated. In the demographic context of a growing population of older adults, this raises many potential health concerns, including resilience to extreme heat events in a changing climate. Furthermore, the heat-related risks due to age and social isolation can be exacerbated by other conditions with higher prevalence among older and more socially isolated populations, such as low income and poor housing (Kilbourne 2002; Lubik and Kosatsky 2019; Yardley et al. 2011). Indeed, socially isolated older adults are less likely to have access to air conditioning, the most effective intervention during extreme heat events (Kilbourne 2002; Yardley et al. 2011).

## Health protection during extreme heat events

Interventions to reduce the health impacts of heat exposure have been well described and may be applied at the individual, household, and community levels (Hajat et al. 2010; Nurse et al. 2010). During heat events, individuals are encouraged to wear light and loose-fitting clothing, seek cool spaces, limit exertion, and stay hydrated; households will benefit from air conditioning, window shades, and fans; communities can incorporate health-protecting designs such as cooling centres, accessible shade, and water features that provide refuge and mitigate the urban heat island effect. With increasing numbers of hot days predicted, these individual and population approaches to extreme heat events can mitigate health impacts of climate change. However, barriers may exist to the uptake and effectiveness of these interventions, with social determinants of health affecting material resources, use of public spaces, and access to transportation (Sampson et al. 2013).

## Social connection as intervention during extreme heat events

The importance of social connection in health promotion has been explored in many areas, including healthy eating, physical activity, mental health, and substance use (Iacoviello and Charney 2014; Petrasek MacDonald et al. 2015). Social connection should also be considered among the tools available for climate change adaptation. This includes the development of greater connection within communities to build overall resilience, and strategies specific to extreme heat events. Public health agencies, municipal governments, and community-based organizations have increasingly recognized the role of social isolation as a risk factor during hot weather, as well as the opportunity to use or build social connection as a protective response. In terms of public health communication, key messages have focused on individual and community imperatives to check in with people who may otherwise be isolated (Lubik and Kosatsky 2019).

More proactively, government and community agencies have an opportunity during pre-season planning to identify potentially isolated residents, clients, and patients receiving health and social services such as home care and food delivery. Pre-existing responder awareness of at-risk communities and individuals will allow for more timely provision of supports during extreme heat events. For example, the Montreal Heat Response Plan (MHRP) recommends an initial screening by healthcare providers to identify clients who are vulnerable to hot weather, including the socially isolated (Price et al. 2013). These clients receive intensified interventions for health protection during heat events, such as more frequent phone calls and visits to check on well-being and uptake of preventive advice (Price et al. 2013). In a follow-up assessment of the MHRP, data collated from reports, questionnaires, and focus groups found that increased contact with vulnerable clients was the most widely implemented recommendation, but that annual updating of vulnerability lists was the most widely cited challenge (Price et al. 2018).

Ideally, practice-based interventions at the intersection of heat and isolation can be implemented without adding burden or complexity to existing workloads. One potential example comes from Rome, where practitioners providing in-home services for at-risk older adults have been effective at reducing mortality during extreme heat events (de’Donato et al. 2018). Such practitioners could easily leave hangers on the doors of consenting clients to publicly identify vulnerable community members, though further work would be needed to evaluate the effectiveness of such an approach. Another approach comes from the BC Housing best practice guidelines, developed to provide support to non-profit housing societies across the province (BC Non-Profit Housing Association 2019). The guidelines include place-based interventions to address the intersection of heat and isolation, such as the establishment of a common “chill room” where residents can seek refuge while engaging socially with others. Enhanced social activity in air-conditioned spaces would address one of the major challenges identified by the MHRP review (Price et al. 2018), and could also improve use of public cooling shelters by vulnerable individuals (Sampson et al. 2013).

Given the complex relationship between social isolation and health, and the general scarcity of resources for preventive interventions in the health sector, intersectoral partnerships including local organizations and networks are crucial. Effective partnerships that can leverage social connection to prevent heat illness might include health services, neighbourhood and building associations, business communities, faith-based groups, and municipal centres such as libraries or swimming pools (Price et al. 2013). The social health of communities would also benefit from interventions to build long-term social capital through community-based interventions such as creating welcoming community designs and intergenerational programming (Murayama et al. 2019). For example, *Building Resilient Neighbourhoods* in Victoria, BC, supports the ability of communities to build and utilize connections for emergency preparedness through “Connect & Prepare” workshops (Building Resilient Neighbourhoods 2019).

Strategies for increasing social connection as a protective response against extreme heat events may also have a wide range of health benefits, including greater overall resilience to acute climate change impacts beyond heat illness, such as floods, forest fires, and windstorms. Emerging evidence points to the increasing mental health burden of generalized climate anxiety (Doherty and Clayton 2011), which may be experienced particularly in areas where the climate is changing more rapidly. While initiatives to increase social connection have a promising role for mitigating the acute and direct impacts of extreme weather events, they can also help to address the chronic and indirect impacts of climate change by building emotional resilience and hope for the future (Berry et al. 2010; Doherty and Clayton 2011).

## Conclusion

Extreme heat events are a growing public health threat as the global climate changes. The health impacts of these events are not exclusively due to temperatures, but rather due to a confluence of biological, environmental, and social vulnerabilities. Social connection is a modifiable protective factor for many health outcomes, and among these, it can play an important role in climate change adaptation by protecting those at greatest risk during extreme heat events. Public health agencies and partners should access the growing literature and documented best practices to apply social connection as a tool for climate change adaptation. Meanwhile, public health organizations must adapt to current and anticipated increases in the frequency and severity of extreme heat events and their impacts on an aging population. In planning and responding to these events, specific attention is warranted for socially isolated older adults. Climate change and social connection are complex topics requiring intersectoral responses, and cooperation among many partners is needed for successful public health action.

## References

[CR1] Arthurson K, Baum S (2015). Making space for social inclusion in conceptualising climate change vulnerability. Local Environment.

[CR2] Austin, S. E., Biesbroek, R., Berrang-Ford, L., Ford, J. D., Parker, S., & Fleury, M. D. (2016). Public health adaptation to climate change in OECD countries. *International Journal of Environmental Research and Public Health, 13*(9). 10.3390/ijerph13090889. 10.3390/ijerph13090889 PMC5036722 27618074

[CR3] BC Non-Profit Housing Association (2019). Preparing for extreme heat and poor air quality events: health impacts (webinar).

[CR4] Berry H, Bowen K, Kjellstrom T (2010). Climate change and mental health: a causal pathways framework. International Journal of Public Health.

[CR5] Building Resilient Neighbourhoods (2019). Connect & prepare: together, we can build the future of emergency preparedness. https://resilientneighbourhoods.ca/connect-prepare/. Accessed 13 Dec 2019.

[CR6] Cheng CS, Campbell M, Li Q, Li G, Auld H, Day N (2008). Differential and combined impacts of extreme temperatures and air pollution on human mortality in south–Central Canada. Part II: future estimates. Air Quality, Atmosphere and Health.

[CR7] de’Donato F, Scortichini M, De Sario M, de Martino A, Michelozzi P (2018). Temporal variation in the effect of heat and the role of the Italian heat prevention plan. Public Health.

[CR8] Doherty TJ, Clayton S (2011). The psychological impacts of global climate change. The American Psychologist.

[CR9] Hajat S, O’Connor M, Kosatsky T (2010). Health effects of hot weather: From awareness of risk factors to effective health protection. Lancet.

[CR10] Iacoviello BM, Charney DS (2014). Psychosocial facets of resilience: Implications for preventing posttrauma psychopathology, treating trauma survivors, and enhancing community resilience. European Journal of Psychotraumatology.

[CR11] Kilbourne EM (2002). Heat-related illness: current status of prevention efforts. American Journal of Preventive Medicine.

[CR12] Klinenberg, E. (2002). *Heat wave: a social autopsy of disaster in Chicago* (1st ed.). Chicago: The University of Chicago Press.

[CR13] Kosatsky T, Henderson SB, Pollock SL (2012). Shifts in mortality during a hot weather event in Vancouver, British Columbia: rapid assessment with case-only analysis. American Journal of Public Health.

[CR14] Lubik, A., & Kosatsky, T. (2019). Is mitigating social isolation a planning priority for British Columbia (Canada) municipalities? http://www.bccdc.ca/Our-Services-Site/Documents/Social_Isolation_Report_17Sept2019.pdf. Accessed 9 July 2019.

[CR15] Murayama, Y., Murayama, H., Hasebe, M., Yamaguchi, J., & Fujiwara, Y. (2019). The impact of intergenerational programs on social capital in Japan: a randomized population-based cross-sectional study. *BMC Public Health, 19*(156). 10.1186/s12889-019-6480-3. 10.1186/s12889-019-6480-3 PMC6364465 30727981

[CR16] National Seniors Council (2017). Who’s at risk and what can be done about it? A review of the literature on the social isolation of different groups of seniors.

[CR17] Nurse J, Basher D, Bone A, Bird W (2010). An ecological approach to promoting population mental health and well-being — a response to the challenge of climate change. Perspectives in Public Health.

[CR18] Petrasek MacDonald J, Cunsolo Willox A, Ford JD, Shiwak I, Wood M (2015). Protective factors for mental health and well-being in a changing climate: perspectives from Inuit youth in Nunatsiavut, Labrador. Social Science & Medicine.

[CR19] Poumadère M, Mays C, Le Mer S, Blong R (2005). The 2003 heat wave in France: dangerous climate change here and now. Risk Analysis.

[CR20] Price, K., Perron, S., & King, N. (2013). Implementation of the Montreal heat response plan during the 2010 heat wave. *Canadian Journal of Public Health, 104*(2), e96–e100. 10.1007/BF03405667 PMC6973896 23618220

[CR21] Price K, Benmarhnia T, Gaudet J, Kaiser D, Sadoine M, Perron S (2018). The Montreal heat response plan: evaluation of its implementation towards healthcare professionals and vulnerable populations. Canadian Journal of Public Health.

[CR22] Sampson N, Gronlund C, Buxton M, Catalano L, White-Newsome J, Conlon K (2013). Staying cool in a changing climate: reaching vulnerable populations during heat events. Global Environmental Change.

[CR23] Yardley J, Sigal RJ, Kenny GP (2011). Heat health planning: the importance of social and community factors. [Article]. Global Environmental Change - Human and Policy Dimensions.

